# Equine Oviductal Organoid Generation and Cryopreservation

**DOI:** 10.3390/mps5030051

**Published:** 2022-06-15

**Authors:** Riley E. Thompson, Mindy A. Meyers, D. N. Rao Veeramachaneni, Budhan S. Pukazhenthi, Fiona K. Hollinshead

**Affiliations:** 1 Department of Clinical Sciences, Colorado State University, Fort Collins, CO 80523, USA; melinda.meyers@colostate.edu (M.A.M.); fiona.hollinshead@colostate.edu (F.K.H.); 2 Department of Biomedical Sciences, Colorado State University, Fort Collins, CO 80523, USA; rao@colostate.edu; 3 Center for Species Survival, Smithsonian National Zoo and Conservation Biology Institute, Front Royal, VA 22630, USA; pukazhenthib@si.edu

**Keywords:** equine, mare, horse, oviduct, fallopian tube, organoid, in vitro, cell culture, cryopreservation

## Abstract

Organoids are a type of three-dimensional (3D) cell culture that more closely mimic the in vivo environment and can be maintained in the long term. To date, oviductal organoids have only been reported in laboratory mice, women, and cattle. Equine oviductal organoids were generated and cultured for 42 days (including 3 passages and freeze–thawing at passage 1). Consistent with the reports in mouse and human oviductal organoids, the equine oviductal organoids revealed round cell clusters with a central lumen. Developing a 3D model of the mare oviduct may allow for an increased understanding of their normal physiology, including hormonal regulation. These organoids may provide an environment that mimics the in vivo equine oviduct and facilitate improved in vitro embryo production in equids.

## 1. Introduction

The oviduct (fallopian tube) is not merely a tube for the transport of sperm, oocytes, and embryos. The oviduct is critical for successful fertilization as the final maturation of oocytes and maintenance of the sperm reservoir to facilitate sperm capacitation as well as fertilization and early embryo development occur in this organ [1]. Both secretory and ciliated epithelial cells are located in the oviduct. The secretory cells contribute to the oviductal fluid and cilia beat rhythmically to transport gametes and embryos through the oviduct [2].

In mares, a conventional in vitro fertilization (IVF) protocol has not been reliable as an artificial reproductive technique (ART), most likely due to the limitations of the extant culture conditions that fail to support equine sperm capacitation [3]. Intracytoplasmic sperm injection (ICSI) has been successful as an alternative to IVF in mares, but ICSI requires specialized equipment and personnel with extensive training. Although the application of ICSI has gained popularity among researchers and practitioners, the utility of this ART remains limited in equine breeding. Creating an in vitro environment that mimics the in vivo oviduct may allow for successful IVF in mares because the oviductal secretions that support sperm capacitation and fertilization would be present. 

Organoids are three-dimensional (3D) cell clusters that are generated from either stem cells or organ progenitor cells that self-assemble and remain genetically and phenotypically stable during a long-term cell culture (months) [4,5]. Organoids are more similar to the in vivo environment than classical monolayer or explant cultures as challenges associated with these two cell culture systems include a lack of normal in vivo structural orientation and an inability to proliferate in the long term, respectively. The development of organoids derived from reproductive tissue has been limited. To date, organoids have been generated using vaginal tissue (mouse) [6], cervical tissue (human and mouse) [7,8], trophoblasts (human) [9], endometrium (human, mouse, and equine) [10,11,12], oviductal tissue (human, mouse, and bovine) [13,14,15,16,17,18,19,20], and ovarian tissue (human and mouse) [15,16,17,21,22]. This is the first report of oviductal organoid generation from mares. 

Equine oviductal organoids may provide an in vitro environment that is more similar to conditions in vivo, which may allow improvements in ARTs such as IVF or ICSI. Additional applications include addressing knowledge gaps in the understanding of normal physiological processes in the mare that are challenging to evaluate in vivo (such as oviductal sperm binding) because the direct visualization of the oviduct in vivo is not straightforward. Our objective was to develop a reliable and repeatable protocol to establish viable organoids in the long term using oviductal tissue from mares. Here, we describe the establishment of mare oviductal organoids utilizing fresh tissue and the re-establishment of oviductal organoids following cryopreservation and thawing. This protocol was developed by adapting and modifying a combination of previously reported culture protocols for human and mouse oviductal organoids and human, mouse, and mare endometrial organoids [10,11,12,13,14]. 

## 2. Experimental Design

### 2.1. Materials

- No. 20 scalpel blade (Hill-Rom Holdings, Chicago, IL, USA; Cat. no.: 371120)
- 100 × 15 mm tissue culture dishes (Fisher Scientific, Waltham, MA, USA; Cat. no.: FB0875713)
- 35 × 10 mm tissue culture dishes (Corning, Corning, NY, USA; Cat. no.: 353001)
- 48-well culture plates (Corning, Corning, NY, USA; Cat. no.: 3548)
- 2 mL cryovials (Light Labs, Aurora, CO, USA; Cat. no.: E-4900)
- 50 mL conical tubes (ThermoFisher Scientific, Waltham, MA, USA; Cat. no.: 339653)
- Microcentrifuge tubes (VWR, Radnor, PA, USA; Cat. no.: 490004-444)
- 12.5, 20, 200, and 1000 µL pipette ClipTip tips (ThermoScientific, Waltham, MA, USA; Cat. no.: 94420043, 94420218, 94420318, and 94420813)
- 40 µm cell strainers (Greiner Bio-One, Monroe, NC, USA; Cat. no.: 542040)
- 100 µm cell strainers (Greiner Bio-One, Monroe, NC, USA; Cat. no.: 542000)
- 20 mL syringes (MWI Animal Health, Aurora, CO, USA; Cat. no.: 670063)
- 0.22 µm syringe filters (Cell Treat, Pepperell, MA, USA; Cat. no.: 229747)
- Ice
- Phosphate-buffered saline (Accuris Life Science Reagents, Edison, NJ, USA; Cat. no.: EB1200)
- MEM Eagle with Earle’s salts (Sigma, St. Louis, MO, USA; Cat. no.: M2279)
- RPMI 1640 (Gibco, Waltham, MA, USA; Cat. no.: 11875-085)
- DMEM/F12 without phenol red with glutamax (Gibco, Waltham, MA, USA; Cat. no.: 21041-025)
- HEPES (Sigma, St. Louis, MO, USA; Cat. no.: H0887)
- Penicillin–streptomycin (Sigma, St. Louis, MO, USA; Cat. no.: P4333)
- Sodium pyruvate (Gibco, Waltham, MA, USA; Cat. no.: 11360-070)
- Glutamax (Gibco, Waltham, MA, USA; Cat. no.: 35050-061)
- Bovine serum albumin (Sigma, St. Louis, MO, USA; Cat. no.: A9418)
- Fetal bovine serum (Peak Serum, Wellington, CO, USA; Cat. no.: PS-FB1)
- B27 Plus (Gibco, Waltham, MA, USA; Cat. no.: A35828-01)
- N2 (Gibco, Waltham, MA, USA; Cat. no.: 17502-048)
- Insulin–transferrin–selenium (Gibco, Waltham, MA, USA; Cat. no.: 41400-045)
- Nicotinamide (Sigma, St. Louis, MO, USA; Cat. no.: N0636)
- Recombinant human EGF (R&D systems, Minneapolis, MN, USA; Cat. no.: 236-EG)
- Recombinant human FGF-10 (PeproTech, Cranbury, NJ, USA; Cat. no.: 100-26)
- Recombinant human Noggin (R&D systems, Minneapolis, MN, USA; Cat. no.: 6057-NG/CF)
- A83-01 (Tocris, Minneapolis, MN, USA; Cat. no.: 2939)
- N-acetyl-L-cysteine (EMD Millipore, Billerica, MA, USA; Cat. no.: 106425)
- Y-27632 (EMD Millipore, Billerica, MA, USA; Cat. no.: 688000)
- SB202190 (Sigma, St. Louis, MO, USA; Cat. no.: S7067)
- Dimethyl sulfoxide (Sigma, St. Louis, MO, USA; Cat. no.: D2650)
- Collagenase V (Sigma, St. Louis, MO, USA; Cat. no.: C9263)
- Dispase II (Sigma, St. Louis, MO, USA; Cat. no.: D4693)
- Matrigel or Cultrex UltiMatrix (phenol red-free, growth factor reduced) (Corning, Corning, NY, USA; Cat. no.: 356231 or Biotechne, Minneapolis, MN, USA; Cat. no.: BME001-05)
- Cell Recovery Solution or Cultrex Organoid Harvesting Solution (Corning, Corning, NY, USA; Cat. no.: 354253 or Biotechne, Minneapolis, MN, USA; Cat. no.: 3700-100-01)
- 4% paraformaldehyde (ThermoFisher Scientific, Waltham, MA, USA; Cat. no.: J19943-K2)
- 2% agarose prepared with water (Bio-Rad, Hercules, CA, USA; Cat. no.: 1613101)
- 70% ethanol.

### 2.2. Equipment

- Surgical scissors (VWR, Radnor, PA, USA; Cat. no.: 63042-002)
- Thumb forceps (VWR, Radnor, PA, USA; Cat. no.: 10198-012)
- Biosafety cabinet (Labconco, Kansas City, MO, USA; Cat. no.: 302411101)
- ThermoMixer C shaking–heating block (Eppendorf, Enfield, CT, USA; Cat. no.: 5382000023)
- 10, 20, 200, and 1000 µL ClipTip pipettes (ThermoScientific, Waltham, MA, USA; Cat. no.: 4641320N, 4641180N, 4641210N, 4641230N)
- Inverted microscope TMS (Nikon, Tokyo, Japan)
- Sorvall Legend Micro 21R Microcentrifuge (ThermoScientific, Waltham, MA, USA; Cat. no.: 75002447)
- Heracell VIOS 160i CO_2_ incubator (ThermoScientific, Waltham, MA, USA; Cat. no.: 51030285)
- Cool Cell (Biocision, San Rafael, CA, USA; Cat. no.: BCS-405)
- Liquid nitrogen and storage tanks (Chart Biomedical, Ball Ground, GA, USA; Cat. no.: MVE XC 47/11)
- Extra-long forceps (Aven Tools, Ann Arbor, MI, USA; Cat. no.: 12022)
- Cryo gloves (Electron Microscopy Sciences, Hatfield, PA, USA; Cat. no.: 71076)
- IsoTemp water bath (Fisher Scientific, Waltham, MA, USA; Cat. no.: S3-705-3).

## 3. Procedure

### 3.1. Generation of Equine Oviductal Organoids

#### 3.1.1. Initial Organoid Establishment

1. Thaw Matrigel/Cultrex in 4 °C before starting (can thaw overnight).
2. Warm a 48-well plate to 37 °C.
3. Prepare the handling medium (see Reagents Setup below; 5 mL per oviduct) and digestion solution (5 mL per oviduct).
4. Transport the equine oviduct in sterile PBS (room temperature for immediate processing or maintained overnight at 4 °C).
5. Place oviduct in a 100 × 15 mm Petri dish with 5 mL handling medium.
6. Cut the oviduct open lengthwise with scissors and scrape the lumen of the oviduct with a scalpel blade to release the cells into the handling medium.
7. Transfer the handling medium with the scraped cells into the microcentrifuge tubes. Discard the remaining oviductal tissue.
8. Centrifuge at 600× *g* for 6 min at room temperature (20–22 °C). Remove and discard the supernatant.
9. Add the digestion solution (see Reagents Setup below) to the cell pellets (1 mL per tube with approximately 5 tubes per oviduct).
10. Place the tubes in the shaking–heating block (37 °C; 1000 rpm) with intermittent pipetting using a 1000 μL pipette for 10–20 min. Periodically check aliquots on an inverted microscope (approximately every 5 min) for dissociation of the oviductal glandular epithelium, which appear as worm-like structures.

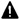

**CRITICAL STEP** Do not over-digest. The oviductal glandular epithelium should be as intact as possible whilst removing the stromal cells and fibrous tissue.

11. Stop the digestion by transferring the tube contents to 50 mL tubes containing RPMI 1640 with 20% fetal bovine serum (20 mL total volume per oviduct).
12. Place a 100 µm cell strainer in a new 50 mL tube and transfer the cell suspension through the cell strainer. Repeat ~4 times with the same strainer.

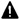

**CRITICAL STEP** Discard the fibrous tissue. This can be removed with thumb forceps.

13. Thoroughly rinse the inverted cell strainer into a 35 × 10 mm Petri dish with 2 mL RPMI 1640.
14. Place a 40 µm cell strainer into the 50 mL tube and transfer the same diluted cell solution through the cell strainer. Repeat ~4 times with the same strainer. (This step will ensure the collection of the smaller glandular fragments).
15. Rinse the inverted cell strainer using the same RPMI 1640 medium that was used for the 100 µm cell strainer.
16. Transfer the glandular suspension from the 35 × 10 mm Petri dish to the microcentrifuge tubes.
17. Centrifuge at 600× *g* for 6 min at room temperature (20–22 °C). Remove and discard the supernatant.
18. Combine the pellets for each oviduct into one centrifuge tube and resuspend in 1 mL DMEM/F12.
19. Centrifuge at 600× *g* for 6 min at room temperature (20–22 °C). Remove and discard the supernatant.
20. Place the cell pellet on ice.
21. Add 20x Matrigel/Cultrex and mix carefully.

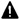

**CRITICAL STEP** Ensure the Matrigel/Cultrex is at 4 °C.

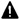

**CRITICAL STEP** Avoid generating bubbles whilst mixing.

**Figure 1:**
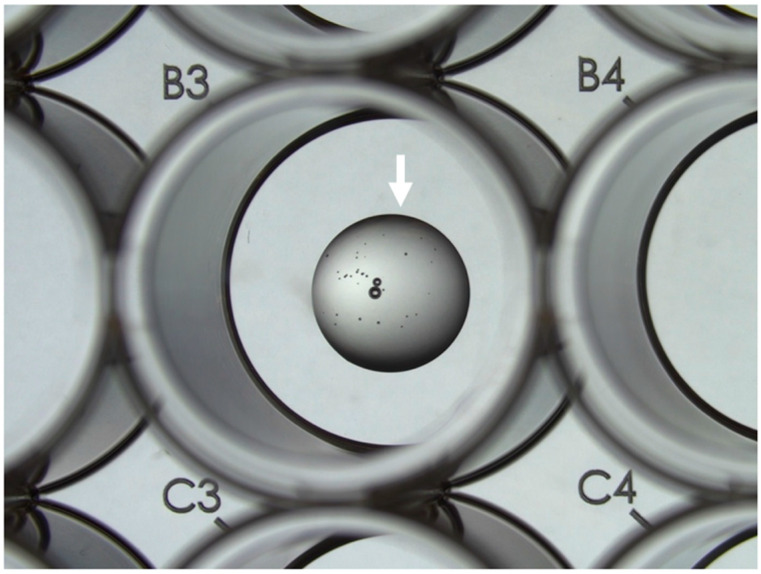
Image of Matrigel/Cultrex droplet (indicated with arrow) in the center of well B3 in a 48-well plate prior to culture medium deposition.

22. Dispense 25 μL droplets into the center of each well in the pre-warmed 48-well plate (Figure 1).
23. Place the plate with the droplets into a 37 °C incubator for 30 min.
24. Add 250 μL organoid medium to each well and place the 48-well plate in an incubator (37 °C, 5% CO_2_).

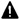

**CRITICAL STEP** Deposit the medium down the side of the well. Do not drop directly onto the Matrigel/Cultrex droplet.

#### 3.1.2. Passage Protocol (Perform This Step Every 7–14 Days Depending on the Growth Rate and Concentration of the Organoids per Well)

1. Thaw Matrigel/Cultrex at 4 °C before starting (can thaw overnight).
2. Warm a 48-well plate to 37 °C.
3. Transfer the well contents to microcentrifuge tubes (4 wells per tube).
4. Centrifuge at 600× *g* for 6 min at room temperature (20–22 °C). Remove and discard the supernatant.
5. Add 200 μL DMEM/F12 to the pellet. Pipette 500 times through a 200 µL pipette tip set at 150 µL.

**OPTIONAL STEP** Pool the pellets at this stage if desired.

6. Add an additional 800 µL DMEM/F12.
7. Centrifuge at 600× *g* for 6 min at room temperature (20–22 °C). Remove and discard the supernatant.
8. Add 200 µL DMEM/F12 to the pellet. Pipette 300 times through a 200 µL pipette tip set at 150 µL.
9. Add an additional 800 µL DMEM/F12.
10. Centrifuge at 600× *g* for 6 min at room temperature (20–22 °C). Remove and discard the supernatant.
11. Add Matrigel/Cultrex and plate as described above.

### 3.2. Cryopreservation and Thawing of Equine Oviductal Organoids

#### 3.2.1. Cryopreservation of the Organoids

1. Remove the culture medium from the wells and replace with 250 µL Cell Recovery Solution (Corning) or Organoid Harvesting Solution (Cultrex) and place the plate on ice for 45–60 min.
2. Transfer the well contents to the microcentrifuge tubes (4 wells per tube).
3. Centrifuge at 600× *g* for 6 min at room temperature (20–22 °C). Remove and discard the supernatant.
4. Add 1 mL DMEM/F12 to the cell pellet.
5. Centrifuge at 600× *g* for 6 min at room temperature (20–22 °C). Remove and discard the supernatant.
6. Combine 4 wells of organoids per 0.25 mL of freezing media (see Reagents Setup below) in a cryovial. Add the freezing medium to the cell pellet dropwise.
7. Place the cryovial into Cool Cell and cool to −80 °C overnight.
8. Transfer the vials to liquid nitrogen the following day and store the cryovials in liquid nitrogen.

#### 3.2.2. Thawing the Organoids

1. Remove the cryovial from the liquid nitrogen tank.
2. Thaw the vial for 1 min in air and then 1 min in a 25 °C water bath [23].
3. Add 5 mL of a thawing medium (see Reagents Setup below) dropwise and gently mix for 10 min.
4. Transfer the cell suspension to a 15 mL centrifuge tube.
5. Centrifuge at 100–200× *g* for 10 min at room temperature (20–22 °C).
6. Remove the supernatant and discard it.
7. Add Matrigel/Cultrex and plate as described above.

### 3.3. Histology

1. Remove the culture media from the wells leaving only the Matrigel droplets.
2. Add 250 μL Cell Recovery Solution (Corning) or Organoid Harvesting Solution (Cultrex) to each well. Place the 48-well plate on ice for 45–60 min.
3. Transfer the contents of the wells to the microcentrifuge tubes.
4. Centrifuge at 600× *g* for 6 min at room temperature (20–22 °C). Remove and discard the supernatant.
5. Resuspend the pellet in 1 mL PBS.

**OPTIONAL STEP** Pool the pellets at this stage if desired.

6. Centrifuge at 600× *g* for 6 min at room temperature (20–22 °C). Remove and discard the supernatant.
7. Add 500 μL of 4% paraformaldehyde without disrupting the pellet. Incubate at room temperature (20–22 °C) for 30 min.
8. Remove and discard the paraformaldehyde.
9. Add 50 μL of warm 2% agarose to the cell pellet.
10. Pick up the whole pellet in agarose with a 200 μL pipette tip and transfer to a Petri dish to solidify as a droplet.

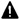

**CRITICAL STEP** Cut the tip off the pipette before transferring the agarose and the cell pellet.

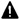

**CRITICAL STEP** Move quickly as the agarose will begin to cool.

11. Transfer the solidified agarose droplet to a microcentrifuge tube containing 70% ethanol until processing for embedding.
12. Routinely embed in paraffin wax, section, and stain with hematoxylin and eosin or a periodic acid-Schiff reagent and hematoxylin.

### 3.4. Transmission Electron Microscopy (TEM)

1. Remove the culture media from the wells leaving only the Matrigel droplets.
2. Add 250 μL Cell Recovery Solution (Corning) or Organoid Harvesting Solution (Cultrex) to each well. Place the 48-well plate on ice for 45–60 min.
3. Transfer the contents of the wells to the microcentrifuge tubes.
4. Centrifuge at 600× *g* for 6 min at room temperature (20–22 °C). Remove and discard the supernatant.
5. Resuspend the pellet in 1 mL PBS.

**OPTIONAL STEP** Pool the pellets at this stage if desired.

6. Centrifuge at 600× *g* for 6 min at room temperature (20–22 °C). Remove and discard the supernatant.
7. Fix the organoid pellet in 500 μL of 2% glutaraldehyde and 4% paraformaldehyde in 0.1 M cacodylate buffer prior to routine TEM sample preparation and imaging.

## 4. Expected Results

**Figure 2:**
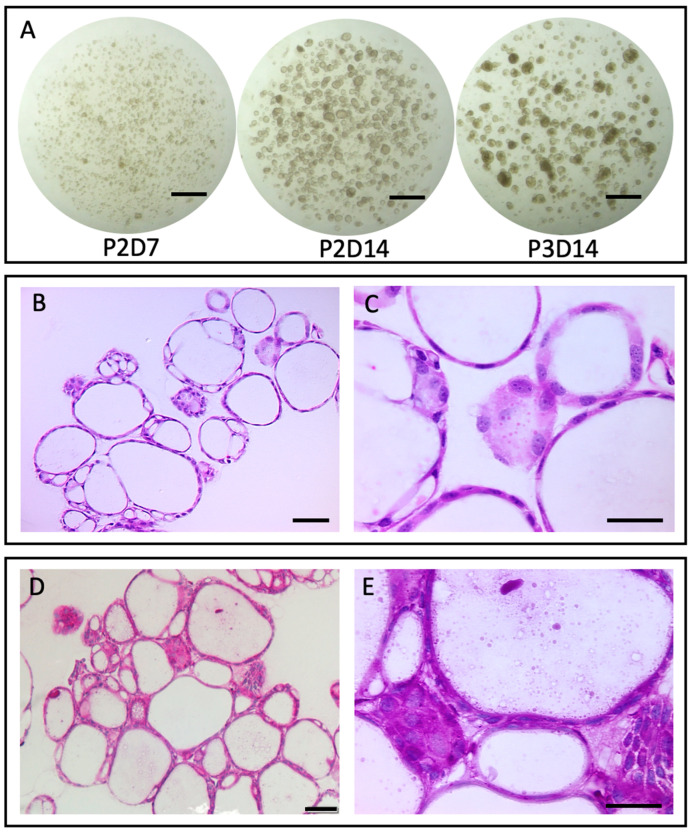
Organoids derived from mare oviductal tissue. Representative culture images of organoids derived from frozen–thawed organoids (frozen at passage 1, day 8; P1D8) at P2D7, P2D14, and P3D14 (**A**) that were cultured for a total of 42 days. Images in the lower panels display histology of frozen–thawed organoids from P2D14 stained with hematoxylin and eosin (**B**,**C**) and with periodic acid-Schiff (PAS) reagent and hematoxylin (**D**,**E**). Pink staining with PAS is indicative of mucin production. Scale bars: 1 mm (**A**); 50 μm (**B**,**D**); and 25 μm (**C**,**E**).

**Figure 3:**
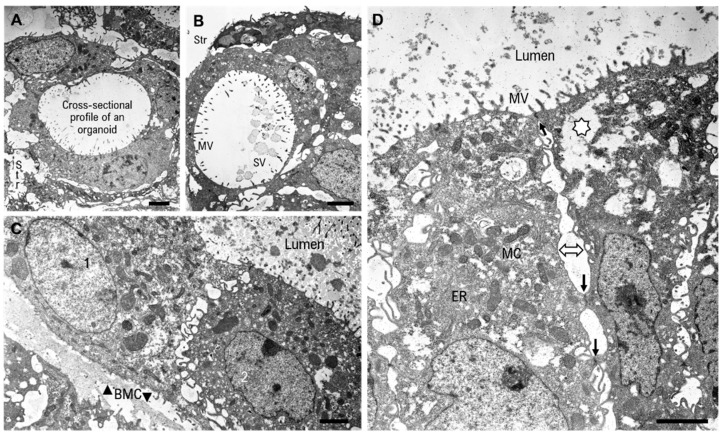
Transmission electron micrographs of cryopreserved organoids (frozen at P1D8 and then cultured until P3D6). (**A**) Three contiguous epithelial cells are seen in this cystic structure surrounded by several stromal (Str) elements. (**B**) Another organoid with discernible microvilli (MV) on the apical surface and luminal secretory vesicles (SV). (**C**) Two conspicuously different morphological phenotypes (numbered 1 and 2), likely indicating a distinct functional differentiation, are seen in this polarized organoid with surface specializations. Note the presence of structurally homogenous amorphous material in the basal aspect, indicating potential basement membrane components (BMC) along with microvilli and secreted material in the apical aspect (lumen). (**D**) In this sagittal section, compared with the epithelial cell on the left, the ultrastructure appears normal, exhibiting stacks of endoplasmic reticulum (ER) and mitochondria (MC) with discernible cristae. The apical portion of the cell on the right manifests discrete irregular cytoplasmic vacuolations (indicated by the octagonal star) and clumping of cellular organelles resulting from disrupted cytoskeletal filaments. These artifacts could be a consequence of freeze–thaw injury. This effect is also obvious in the widened intercellular space (double-headed open arrow). Despite this damage, the intercellular junctions (pointed at by three arrows) appear intact between the normal and partially damaged cell. Scale bar: 2 μm (**A**–**D**).

The above methods resulted in organoid generation, utilizing: (i) fresh equine oviducts collected post mortem during a transitional stage between winter anestrus and cycling utilizing cells from the length of the oviduct (infundibulum, ampulla, and isthmus); and (ii) frozen–thawed organoids at passage 1, day 8 (P1D8), then culturing for an additional four weeks to P3D14 (Figure 2A). The organoid histology (Figure 2B,C) and TEM (Figure 3A) revealed round, cystic structures with apical polarity toward the lumen of the organoids that exhibited microvilli, consistent with the reports of mouse and human oviductal organoids and equine endometrial organoids [12,13,14,24]. Periodic acid-Schiff and hematoxylin (PAS&H) staining (Figure 2D,E) and TEM (Figure 3B,C) confirmed the secretory capability of the organoids. These results demonstrate, for the first time, that oviductal organoids can be established utilizing oviductal tissue from the mare.

The generation of oviductal organoids has been previously reported utilizing oviductal tissue from mice and women [13,14]. Descriptions of oviductal spheroids, another type of 3D cell culture, have been reported [25], but these structures cannot self-assemble or proliferate in the long term and often have a central core of necrosis that may negatively impact the cell culture outcome [26,27]. Furthermore, preliminary data of organoids derived from bovine oviductal tissue have recently been reported in two review articles [19,20]. 

Mouse oviductal organoids demonstrated a variability in organoid growth among the segments with the infundibulum displaying the greatest growth rate [13]. In the current study, we utilized cells from the entire length of the mare oviduct rather than isolating the functional segments of the oviduct. Future studies of equine oviductal organoids should consider an evaluation of the organoids generated from specific oviductal segments. 

Although the co-culture of equine oviductal organoids with gametes and/or embryos may improve ARTs, most organoids are generated utilizing an extracellular matrix such as Matrigel^®^, which may affect the direct contact of the gametes and/or embryos with the organoids. An alternative method to improve ARTs may utilize extracellular vesicles (EVs) secreted by oviductal organoids. A method for isolating secretions from the lumen of human endometrial organoids has been reported that utilized centrifugation to disrupt the organoids without affecting the cellular viability [28], which could be utilized for EV isolation from the lumen of the organoids and compared with EVs secreted by the basal aspect of the organoids into the conditioned medium.

Extracellular vesicles are nanoparticles that contain nucleic acids and proteins within a lipid bilayer and are naturally secreted by cells to facilitate intercellular communication [29]. EVs produced in vivo have demonstrated a beneficial impact on ARTs. EVs in cow follicular fluid are associated with the modulation of the arrest of oocyte meiosis [30]. Furthermore, cat oocytes vitrified with follicular fluid EVs positively impacted the resumption of meiosis in frozen–thawed oocytes [31]. EVs isolated from oviductal fluid impact sperm viability, sperm motility, the formation of the sperm reservoir, oocyte maturation, sperm–oocyte binding, fertilization, and embryo development and quality [25,32,33,34,35,36,37,38]. Furthermore, EVs isolated from cow uterine fluid supplemented in an in vitro culture medium improved the somatic cell nuclear transfer embryonic development and blastocyst quality [39]. These reports indicate a benefit to gamete and embryo production when co-incubated with EVs from the reproductive tract. 

However, the collection of in vivo-derived EVs is not practical, sustainable, or repeatable. Therefore, an in vitro source of oviductal cell-derived EVs that is physiologically similar to the in vivo oviductal environment—such as EVs produced by oviductal organoids—would facilitate advancements in many ARTs. A novel approach may be to isolate the EVs that the organoid cells produce for co-incubation with equine gametes and/or embryos. Although additional research is required to evaluate the functional outcome, EVs secreted by equine oviductal organoids may provide an in vitro environment that can closely simulate the in vivo oviduct. This may facilitate improvements in ARTs, including IVF in equids.

The 3D organoid culture methods described here provide a roadmap to improve our understanding of equine oviductal physiology and the potential to make significant advances in equine ARTs to improve equine reproductive efficiency.

## 5. Reagent Setup

For all items below, combine the components and then filter-sterilize using syringe filters.

Handling medium [23]:
  MEM with Earle’s salts.
  HEPES (25 mM).
  Penicillin (100 U/mL).
  Streptomycin (0.1 mg/mL).
  Pyruvate (0.1 mM).
  Glutamax (2 mM).
  Bovine serum albumin (5% *v*/*v*).
Digestion solution [12]:
  RPMI 1640.
  Collagenase V (0.4 mg/mL).
  Dispase II (1.25 U/mL).
Organoid medium:
  DMEM/F12 without phenol red with glutamax.
  Penicillin/streptomycin (1%).
  B27 Plus (2%).
  N2 (1%).
  Insulin–transferrin–selenium (1%).
  Nicotinamide (1 mM).
  Recombinant human EGF (50 ng/mL).
  Recombinant human FGF-10 (50 ng/mL).
  Recombinant human Noggin (100 ng/mL).
  TGFβ/Alk inhibitor A83-01 (0.5 µM).
  N-acetyl-L-cysteine (1.25 mM).
  SB202190 (10 µM).
  Y27632 (10 µM).
Freezing medium [23]:
  MEM with Earle’s salts.
  Dimethyl sulfoxide (10%).
  HEPES (25 mM)
  Penicillin (100 U/mL).
  Streptomycin (0.1 mg/mL).
  Pyruvate (0.1 mM).
  Glutamax (2 mM)
  Fetal bovine serum (20% *v*/*v*).
Thawing medium [23]:
  MEM with Earle’s salts.
  HEPES (25 mM).
  Penicillin (100 U/mL).
  Streptomycin (0.1 mg/mL).
  Pyruvate (0.1 mM).
  Glutamax (2 mM).
  Fetal bovine serum (20% *v*/*v*).

## Data Availability

The data presented in this study are available within the body of the article.
